# Correlation Between Brain Structure Atrophy and Plasma Amyloid-β and Phosphorylated Tau in Patients With Alzheimer’s Disease and Amnestic Mild Cognitive Impairment Explored by Surface-Based Morphometry

**DOI:** 10.3389/fnagi.2022.816043

**Published:** 2022-04-25

**Authors:** Kaidi Li, Hang Qu, Mingyi Ma, Chenyu Xia, Ming Cai, Fang Han, Qing Zhang, Xinyi Gu, Qiang Ma

**Affiliations:** ^1^Department of Neurology, Affiliated Hospital of Inner Mongolia Medical University, Hohhot, China; ^2^Department of Imaging, Yangzhou First People’s Hospital, Affiliated Hospital of Yangzhou University, Yangzhou, China; ^3^Department of Molecular and Cellular Biology, University of Illinois Urbana-Champaign, Urbana, IL, United States; ^4^Department of Neurology, Affiliated Zhongshan Hospital of Dalian University, Dalian, China; ^5^Department of Imaging, Affiliated Zhongshan Hospital of Dalian University, Dalian, China

**Keywords:** plasma Aβ, amnestic mild cognitive impairment, Alzheimer’s disease, surface-based morphometry, plasma Tau

## Abstract

**Objective:**

To investigate the changes in the cortical thickness of the region of interest (ROI) and plasma Aβ40, Aβ42, and phosphorylated Tau (P-Tau) concentrations in patients with Alzheimer’s disease (AD) and amnestic mild cognitive impairment (aMCI) as the disease progressed with surface-based morphometry (SBM), to analyze the correlation between ROI cortical thickness and measured plasma indexes and neuropsychological scales, and to explore the clinical value of ROI cortical thickness combined with plasma Aβ40, Aβ42, and P-Tau in the early recognition and diagnosis of AD.

**Methods:**

This study enrolled 33 patients with AD, 48 patients with aMCI, and 33 healthy controls (normal control, NC). Concentration changes in plasma Aβ42, Aβ40, and P-Tau collected in each group were analyzed. Meanwhile, the whole brain T1 structure images (T1WI-3D-MPRAGE) of each group of patients were collected, and T1 image in AD-aMCI, AD-NC, and aMCI-NC group were analyzed and processed by SBM technology to obtain brain regions with statistical differences as clusters, and the cortical thickness of each cluster was extracted. Multivariate ordered logistic regression analysis was used to screen out the measured plasma indexes and the indexes with independent risk factors in the cortical thickness of each cluster. Three comparative receiver operating characteristic (ROC) curves of AD-aMCI, AD-NC, and aMCI-NC groups were plotted, respectively, to explore the diagnostic value of multi-factor combined prediction for cognitive impairment. The relationship between cortical thickness and plasma indexes, and between cortical thickness and Mini-Mental State Examination (MMSE) and Montreal Cognitive Assessment (MoCA) scores were clarified by Pearson correlation analysis.

**Results:**

Plasma Aβ40, Aβ42, and P-Tau proteins in the NC, aMCI, and AD groups increased with the progression of AD (*P* < 0.01); cortical thickness reductions in the AD-aMCI groups and AD-NC groups mainly involved the bilateral superior temporal gyrus, transverse temporal gyrus, superior marginal gyrus, insula, right entorhinal cortex, right fusiform gyrus, and cingulate gyrus. However, there were no statistical significances in cortical thickness reductions in the aMCI and NC groups. The cortical thickness of the ROI was negatively correlated with plasma Aβ40, Aβ42, and P-Tau concentrations (*P* < 0.05), and the cortical thickness of the ROI was positively correlated with MMSE and MoCA scores. Independent risk factors such as Aβ40, Aβ42, P-Tau, and AD-NC cluster 1R (right superior temporal gyrus, temporal pole, entorhinal cortex, transverse temporal gyrus, fusiform gyrus, superior marginal gyrus, middle temporal gyrus, and inferior temporal gyrus) were combined to plot ROC curves. The diagnostic efficiency of plasma indexes was higher than that of cortical thickness indexes, the diagnostic efficiency of ROC curves after the combination of cortical thickness and plasma indexes was higher than that of cortical thickness or plasma indexes alone.

**Conclusion:**

Plasma Aβ40, Aβ42, and P-Tau may be potential biomarkers for early prediction of AD. As the disease progressed, AD patients developed cortical atrophy characterized by atrophy of the medial temporal lobe. The combined prediction of these region and plasma Aβ40, Aβ42, and P-Tau had a higher diagnostic value than single-factor prediction for cognitive decline.

## Introduction

Alzheimer’s disease (AD) is a neurodegenerative disease characterized by progressive cognitive and behavioral disorders. Mild cognitive impairment (MCI) is an intermediate stage between AD and normal aging. Amnestic mild cognitive impairment (aMCI) is a major type of MCI. In the aMCI stage, approximately half of the patients may be converted to AD patients (Petersen et al., 1999).

At present, it is believed that amyloid-β (Aβ) deposition and Tau neuron damage in the brain are the main causes of AD (Jack et al., 2013; Liu et al., 2019). The new drug aducanumab, which has recently been approved by the Food and Drug Administration (FDA) for the treatment of AD, targets to clear Aβ plaques in the brain (Sevigny et al., 2016; Schneider, 2020) and improve the cognitive function of AD patients. The Aβ and Tau in cerebrospinal fluid (CSF) has been used for the diagnosing AD and aMCI (Sperling et al., 2011; Dubois et al., 2014). However, CSF needs to be obtained through an invasive lumbar puncture, patient compliance is lower, and the lumbar puncture increases the chance of iatrogenic infection. By contrast, blood is easy to obtain. Current studies have found that changes in the concentrations of Aβ and Tau in the peripheral blood are closely related to the clearance and transport of Aβ and Tau in the central nervous system (Tarasoff-Conway et al., 2015). Palmqvist et al. (2019) found that CSF and plasma biomarkers of AD patients changed almost simultaneously. The study of Lue et al. (2017) revealed that compared with healthy older adults, aMCI patients had changes in their plasma Aβ42 and Tau levels and could reflect the progression of AD. As mentioned above, a large number of studies on the changes of plasma Aβ and Tau concentrations in patients with aMCI and early AD have confirmed that changes in plasma Aβ and Tau concentrations can reflect changes in brain Aβ and Tau concentrations to a certain extent.

Changes in the brain structure of AD patients are currently recognized research results. In the early stages of AD, specific brain regions such as the hippocampus, entorhinal cortex, and amygdala have begun to shrink (Horínek et al., 2007; Tang et al., 2015; Maass et al., 2018). The degree of atrophy is related to the progression of dementia (Dubois et al., 2007; Pettigrew et al., 2016; Marquié et al., 2017; Moscoso et al., 2019). At present, the measurement of cortical thickness by surface-based morphometry (SBM) has been increasingly used in the research of evaluating the degree of brain atrophy, which can evaluate the atrophy region in the whole brain more accurately and specifically. Previous studies on the cortical thickness of AD patients uncovered that AD patients had a reduction in cortical thickness in specific brain regions in contrast to normal people (Pettigrew et al., 2017; Racine et al., 2018; Weise et al., 2019). Sun et al. (2019) adopted SBM technology to analyze the thickness of the cerebral cortex in AD patients and normal elderly people, and found that the cortical thickness of the fusiform gyrus, middle frontal gyrus, cingulate gyrus, posterior orbital gyrus, insula, caudate nucleus, superior frontal gyrus, and inferior parietal lobule of AD patients was significantly reduced compared with normal elderly people. Racine et al. (2018) also analyzed the cortical thickness of normal aging and AD patients with SBM technology, and illustrated that the reduction of cortical thickness in AD patients mainly occurred in the medial temporal lobe, inferior temporal gyrus, temporal pole, superior parietal lobule, supramarginal gyrus, angular gyrus, superior frontal gyrus, precuneus, and other parts. These studies refered above have suggested that these brain regions are closely associated with cognition.

Present studies have verified that in the process of AD, Aβ deposition, and phosphorylated Tau (P-Tau) neuron damage have begun to appear at first in the brain, which further leads to brain atrophy (Sperling et al., 2011). Meanwhile, changes in plasma Aβ and Tau concentrations can reflect changes in brain Aβ and Tau concentrations to some extent. We further hypothesized that changes in plasma Aβ and Tau concentrations in AD patients were related to changes in brain structure, and the combination of cortical thickness indexes and plasma indexes could better predict AD.

## Materials and Methods

### Study Population

A total of 112 inpatients in the Department of Neurology, Affiliated Zhongshan Hospital of Dalian University were enrolled in this study from February 2018 to December 2019, all of whom were right-handed. Of these patients, 31 patients suffered from mild and moderate AD, including 13 males and 18 females, with an average age of 74.71 years, and all the disease courses of more than 2 years. There were 48 patients with aMCI, including 20 males and 28 females, with an average age of 68.54 years, and all the disease courses of over 3 months. There were 33 normal elderly controls, including 21 males and 12 females, with an average age of 66.21 years.

### Inclusion and Exclusion Criteria

All enrolled patients completed the Activity of Daily Living (ADL) Scale, Clinical Dementia Rating (CDR) Scale (Berg, 1988; Morris, 1993), Hamilton Anxiety Scale (HAMA) (Hamilton, 1959), Hamilton Depression Scale (HAMD) (Hamilton, 1960), Mini-Mental State Examination (MMSE) (Folstein et al., 1975), Montreal Cognitive Assessment (MoCA) (Nasreddine et al., 2005), and other scale evaluations. In addition, the enrolled patients were required to complete the Boston Naming Test (BNT), Tandem Mass Tag (TMT), Clock-Drawing Test (CDT), Auditory Verbal Learning Test, Huashan Version (AVLT-H) (Zhao et al., 2015), Wechsler Memory Scale Visual Reproduction (WMS-VR), and other scales to test language, executive function, visual space function, memory function, and other fields. Scale assessors were all experienced neurologists who received professional neuropsychological scale training. The above scale evaluation processes were carried out in a quiet and undisturbed environment.

#### Amnestic Mild Cognitive Impairment Group

This study mainly adopted the diagnostic criteria of Peterson in 2004 (Petersen, 2004) and the definition of aMCI by the MCI Working Group of the European Consortium on Alzheimer’s Disease in 2006 (Portet et al., 2006). The inclusion criteria for the aMCI group were as follows: (1) the patient had a main complaint of memory deficits, which was confirmed by an informant; (2) there was objective evidence of memory deficits. Sub-items to assess memory such as immediate memory and delayed recall in the MMSE and MoCA were scored in this study. The sub-score of less than 1.5 SD of the published normative values for age and/or educational was used as objective evidence of memory deficits, with the total score of 18 ≤ MMSE ≤ 26 and 20 ≤ MoCA ≤ 26; (3) ADL was not impaired (4) the AD diagnostic criteria were not met; the criteria for suspicious dementia were met and the criteria for dementia were not yet met according to clinical dementia rating (CDR) scale, with CDR = 0.5 points. The neuropsychological measurement of at least 1.5 SD lower than the published normative values for age and/or education indicated cognitive impairment. Exclusion criteria were: (1) those who were unable to cooperate with relevant inspections and tests in the study; (2) those who had contraindications to magnetic resonance imaging (MRI) examination; (3) related diseases that may cause cognitive impairment, such as frontotemporal dementia, dementia with Lewy bodies, vascular dementia, Parkinson’s disease with dementia, and other types of dementia; mental disorders; thyroid disease, folic acid deficiency, vitamin B_12_ deficiency, severe anemia and other endocrine, and metabolic diseases; and cognitive impairment caused by trauma, infection, drugs, or alcoholism, etc.

#### Alzheimer’s Disease Group

This screening met the diagnostic criteria issued by the National Institute on Aging (NIA) and the Alzheimer’s Association (AA) in 2011 (Jack et al., 2011) and referred to the “Diagnostic and Statistical Manual of Mental Disorders” (DSM-IV-R) diagnostic criteria (Arthur et al., 2001). Inclusion criteria: the decline of memory function or other cognitive domain functions objectively existed, with MMSE < 20 points and MoCA < 18 points; ADL was impaired (ADL ≥ 22 points); CDR = 1 point; except for changes in AD, there were no other abnormalities. The exclusion criteria were the same as those in the aMCI group.

#### Normal Control Group

The normal elderly who were in the same period, aged 50–75 years, and whose gender, handedness, and education level matched those of the aMCI group and AD group were screened and enrolled from the memory outpatient department of neurology specialists were selected as the control group (normal control, NC). Inclusion criteria: no MRI examination contraindications, no abnormalities shown by head MRI, no memory deficits or other cognitive decline performance, and no mental or neurological diseases; MMSE ≥ 26 and MoCA ≥ 26. The exclusion criteria were the same as those in the aMCI group.

### Clinical Data Collection

#### General Clinical Data Collection

This study was approved by the Ethics Committee of the Affiliated Zhongshan Hospital of Dalian University (approval number: 2019142). All the subjects signed informed consent forms for this study. The general data of subjects, such as age, gender, handedness, past medical history, family history and genetic history, education level, history of memory deficits, and results of nervous system examination, were collected to rule out nervous system diseases and other systems-related diseases that may cause cognitive decline.

#### Laboratory Examinations

In the early morning, the venous blood of subjects was collected under fasting conditions for examinations of blood routine, coagulation function, blood lipids, blood sugar, liver and kidney function, thyroid function, myocardial enzyme spectrum, homocysteine, tumor markers, hepatitis, acquired immunodeficiency syndrome (AIDS), syphilis, and other laboratory examinations, to exclude dementia induced by infectious factors such as central nervous system infections caused by pathogens such as *Treponema pallidum* and human immunodeficiency virus (HIV), as well as cognitive decline caused by metabolic factors such as diabetic encephalopathy and Hashimoto’s encephalopathy, paraneoplastic limbic encephalitis caused by tumors and other factors, and cognitive decline caused by other systemic diseases.

### Blood Sample Collection and Test

A double antibody sandwich enzyme-linked immunosorbent assay (ELISA) was used to determine the plasma levels of Aβ42, Aβ40, and P-Tau. The phosphorylation sites in P-Tau detection were Ser396 and Ser404. The kit used in this assay was provided by Wuhan Fine Biotech Co., Ltd., China. The whole process was conducted in strict accordance with the reagent instructions. The specific steps included the following: venous blood was collected and placed in an EDTA anticoagulation tube, and centrifuged at 2000 r/min for 10 min under 4°C. Then the supernatant was collected and placed it in an EDTA anticoagulation tube, and refrigerated at −80°C (the whole process was completed within 2 h). Test samples and the kit were put at room temperature for 30 min, and protease inhibitors were added to prevent various proteases from degrading Aβ and P-Tau. According to concentration gradients, 6 SD of double-reduced concentration were prepared, and 100 μL of prepared standards of each concentration and the test samples were added to the microwell plate coated with Aβ42 (Aβ40 and P-Tau) specific antibodies, so that Aβ42 (Aβ40 and P-Tau) in the standard samples and the samples was combined with immobilized antibodies, and incubated, followed by plate washing. Subsequently, 100 μL of biotin-labeled detection antibodies were added to each well on the ELISA plate and incubated. After the samples were washed, antibiotin and horseradish peroxidase (HRP) were added to the microwell. After the samples were washed again, chromogenic substrates were added for chromogenic reactions, and finally the chromogenic reactions were terminated. The optical density (OD) of each well was measured with a microplate reader at 450 nm. Later, a standard curve fitting equation of Aβ42 (Aβ40 or P-Tau) was established, and the concentration of each sample was calculated.

### Magnetic Resonance Imaging Parameters

The MRI scan applied a Siemens superconducting magnetic resonance scanner (3.0 T Magnetom Verio) with a 12-channel standard head coil. Three-dimensional magnetization-prepared rapid acquisition with gradient echo (3D-MPRAGE) imaging sequence scan of the whole brain (from the top of the skull to the foramen magnum) was performed on the enrolled subjects, to obtain a three-dimensional T1 weighted imaging (3D-T1WI) structural image of the whole brain. The scanning parameters were as follows: echo time (TE) = 2.22 ms, repetition time (TR) = 2530 ms, flip angle (FA) = 7°, matrix = 224 × 224, filed of view (FOV) = 224 mm × 224 mm, scanning time: 5 min, slice thickness = 0.9 mm, slice gap = −1 mm, and slice number = 176 layers.

### Surface-Based Morphometry Technology Image Processing

The data processing of this study required the installation of SPM12-v7771 software^1^ and CAT12.7-Beta (r1615) toolbox^2^ in the MATLAB2018 operating environment.^3^

For the SBM technology to estimate cortical thickness, the automatic surface preprocessing algorithm in the CAT12 toolbox was applied. This algorithm reconstructed the central surface of the left and right hemispheres by using a projection-based thickness calculation method, utilized a tissue segmentation to estimate the white matter (WM) distance, and then projected the local maxima (equal to the cortical thickness) to other gray matter voxels by using the adjacent relationship described by the WM distance to determine the cortical thickness (Dahnke et al., 2013). The specific operations were as follows: the projection-based thickness was used to estimate the surface and thickness of the research object, and the central cortical surface of the left and right hemispheres were created. Afterward, a 15 mm half-height half-width smoothing kernel was employed to smooth surface data. The thickness of the whole brain cortex was extracted for comparison, and a statistical model was established.

### Statistical Analysis

SPSS 25.0 software (IBM, Armonk, NY, United State) was adopted for statistical analysis on the general data of the AD, aMCI, and NC groups such as age, gender, education level, MMSE score, and MoCA score. Measurement data were subjected to one-way analysis of variance (ANOVA) and expressed as $\bar{x}$ ± s, and enumeration data were subjected to the Chi-square test and described as percentages. Differences were statistically significant at *P* < 0.05. Plasma Aβ40, Aβ42, and P-Tau concentrations in each group were analyzed by one-way ANOVA, multiple comparisons between groups were performed by the least significant difference (LSD) method (*P* < 0.05), and the results were expressed as $\bar{x}$ ± s.

In the linear model based on the CAT12 surface, imaging data was statistically analyzed by ANOVA for the cortical thickness estimated by SBM in each group. The threshold of *P* < 0.001 and threshold-free cluster enhancement (TFCE) correction (Salimi-Khorshidi et al., 2011) were applied for multiple comparisons to test inter-group differences. The TFCE was used to correct multiple comparisons at the cluster level, and the family-wise error rate was *P* < 0.05. With Desikan-Killiany 40 Atlas (Desikan et al., 2006) as a template, the cortical thickness values of each cluster with statistical differences were extracted. Pearson correlation analysis was utilized to assess the correlation between the cortical thickness of each cluster and MMSE score, MoCA score, and plasma Aβ40, Aβ42, and P-Tau. In addition, according to the segmentation of template, the cortical thickness of different brain regions with statistical differences in each group were extracted, and the cortical thickness of these brain regions was made into ROC curve according to AD-aMCI group, AD-NC group, and aMCI-NC group, and the brain regions with high diagnostic efficiency for AD were selected (see Supplementary Material for details). According to the severity of the disease, the cortical thickness values of each cluster and plasma Aβ40, Aβ42, and P-Tau in the AD-aMCI and AD-NC groups were used as dependent variables to screen out significant indexes through multivariate ordered logistic regression analysis. According to the above selected indexes, the comparative receiver operating characteristic (ROC) curves of AD-aMCI, AD-NC, and aMCI-NC groups were established, and the combined ROC curves of each predictive index were established to obtain the diagnostic value of multi-factor combined prediction for cognitive dysfunction.

## Results

### General Data Analysis

A total of 112 participants were included in this study, and their demographic and neuropsychological details are shown in Table 1. The three groups of data were analyzed by ANOVA and *post hoc* analysis. The results showed that the AD group, aMCI group, and NC group had no statistically LSD in gender and education level (*P* > 0.05), but age, MMSE score, MMSE delayed recall sub-score, MoCA score, MoCA delayed recall sub-score, AVLT-H-SR sub-score, AVLT-H-LR sub-score, WMS-VR-IR sub-score, WMS-VR-DR sub-score, BNT, TMT-B, CDT, ADL, HAMA, and plasma Aβ40, Aβ42, and P-Tau were statistically different among the three groups (*P* < 0.05). The AD and NC groups, and the AD and aMCI groups had statistical differences in MMSE immediate recall sub-score (*P* < 0.05), while the aMCI and NC groups exhibited no statistical difference. Regarding HAMD scores in the three groups, there was no statistical difference between the AD and aMCI groups (*P* > 0.05).

**TABLE 1:** Demographic and clinical variables of participants.

	AD	aMCI	NC	*F*(χ^2^)	*P*
Sample size	31	48	33	–	–
Sex (male)	13 (41.9)	20 (41.7)	21 (63.6)	4.46	0.11
Age	74.71 ± 5.75	68.54 ± 7.73	66.21 ± 8.09	11.49	0.01*
Education	5.13 ± 2.49	5.85 ± 3.31	4.58 ± 3.61	1.60	0.21
MMSE	18.58 ± 4.00	26.18 ± 2.39	27.61 ± 1.87	97.59	< 0.01*
MMSE-Delayed recall	1.19 ± 0.65	1.98 ± 0.53	2.33 ± 0.48	35.90	< 0.01*
MMSE-Immediate recall	1.23 ± 0.67	2.40 ± 0.50	2.58 ± 0.50	58.36	< 0.01*
MoCA	12.45 ± 3.73	21.29 ± 3.68	25.55 ± 2.46	124.73	< 0.01*
MoCA-Delayed recall	1.12 ± 1.09	3.23 ± 0.78	3.91 ± 0.68	94.01	< 0.01*
AVLT-H-SR	1.74 ± 1.18	4.63 ± 1.55	6.55 ± 1.15	103.09	< 0.001**
AVLT-H-LR	0.84 ± 0.93	3.29 ± 1.09	4.70 ± 1.05	113.51	< 0.001**
WMS-VR-IR	1.84 ± 1.10	5.85 ± 1.38	9.12 ± 1.71	210.77	< 0.001**
WMS-VR-DR	1.00 ± 0.82	4.38 ± 1.23	7.39 ± 1.25	252.42	< 0.001**
BNT	15.32 ± 4.79	20.67 ± 3.86	25.55 ± 1.95	60.31	< 0.001**
TMT-B time	361.19 ± 69.19	213.33 ± 45.46	154.85 ± 26.56	150.64	< 0.001**
CDT	0.87 ± 0.89	3.04 ± 0.80	3.42 ± 0.75	94.74	< 0.001**
ADL	29.94 ± 5.84	6.63 ± 5.95	0.64 ± 1.32	309.11	< 0.001**
CDR	1	0.5	0	–	–
HAMD	1.55 ± 1.54	1.13 ± 1.61	0.36 ± 0.65	6.17	< 0.003*
HAMA	4.61 ± 2.26	3.69 ± 2.25	1.45 ± 1.33	21.05	< 0.001**
P-Tau (pg/ml)	138.63 ± 33.80	88.07 ± 31.45	70.27 ± 27.77	41.93	< 0.001**
Aβ40 (pg/ml)	148.74 ± 57.76	118.59 ± 29.66	102.64 ± 32.29	10.94	< 0.001**
Aβ42 (pg/ml)	162.78 ± 29.84	117.84 ± 38.72	97.36 ± 37.65	27.47	< 0.001**
Aβ40/Aβ42	0.94 ± 0.45	1.15 ± 0.60	1.27 ± 0.81	2.14	0.122

*P < 0.01, **P < 0.001.

### Plasma Aβ40, Aβ42, and Phosphorylated Tau Results

The protein concentrations of plasma Aβ42, Aβ40, and P-Tau in the AD, aMCI, and NC groups were compared. In the AD group, the concentrations of plasma Aβ42, Aβ40, and P-Tau were 162.78 ± 29.84 pg/m, 148.74 ± 57.76 pg/ml, and 138.63 ± 33.80 pg/ml, respectively, and Aβ40/Aβ42 was 0.94 ± 0.45. In the aMCI group, the concentrations of plasma Aβ42, Aβ40, and P-Tau were 117.84 ± 38.72 pg/ml, 118.59 ± 29.66 pg/ml, and 88.07 ± 31.45 pg/ml, respectively, and Aβ40/Aβ42 was 1.15 ± 0.60. In the NC group, the concentrations of plasma Aβ42, Aβ40 and P-Tau were 97.36 ± 37.65 pg/ml, 102.64 ± 32.29 pg/ml, and 70.27 ± 27.77 pg/ml, respectively, and Aβ40/Aβ42 was 1.27 ± 0.81.

**FIGURE 1:**
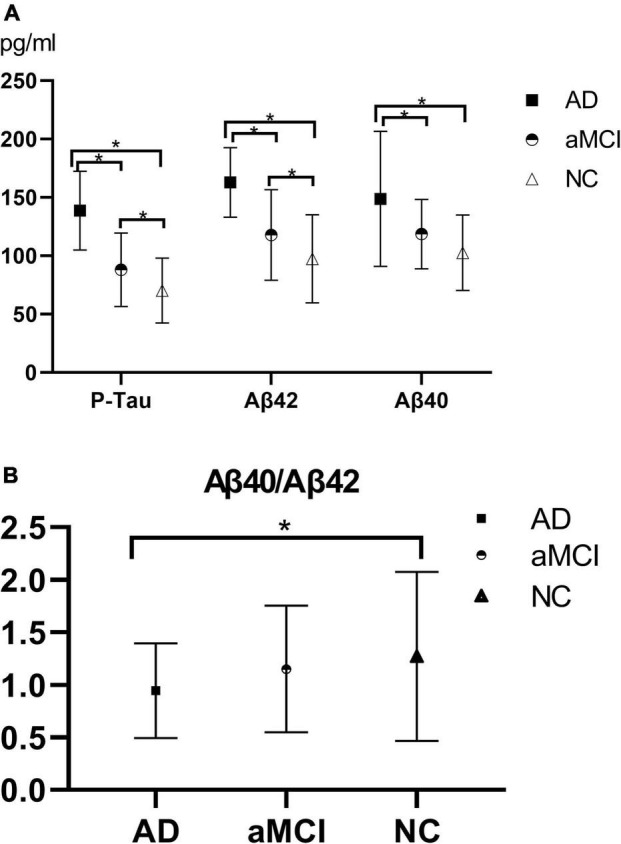
**(A,B)** Histogram of plasma Aβ40, Aβ42, P-Tau, and Aβ40/Aβ42 results in each group. **(A)** The histogram of plasma Aβ40, Aβ42, and P-Tau results in each group illustrated that with the progression of the disease, plasma P-Tau, Aβ42, and Aβ40 all showed upward trends, of which plasma P-Tau, Aβ42, and Aβ40 in the AD-NC groups and AD-aMCI groups exhibited statistical differences (*P* < 0.05); plasma P-Tau and Aβ42 had statistical differences (*P* < 0.05), whereas there was no statistical difference in Aβ40 (*P* > 0.05) between the aMCI and NC groups. **(B)** Aβ40/Aβ42 showed a downward trend as the disease progressed. The AD and NC group demonstrated a statistical difference (*P* < 0.05), while there was no statistical difference between the AD and aMCI groups, and between the aMCI and NC groups (*P* > 0.05). AD, Alzheimer’s disease; aMCI, amnestic mild cognitive impairment; NC, normal control; P-Tau, phosphorylated Tau. *P < 0.05.

Plasma Aβ42, Aβ40, P-Tau, and Aβ40/Aβ42 in each group of AD group, aMCI group, and NC group were subjected to one-way ANOVA, and the results were obtained after multiple comparisons between each group by the LSD test, as shown in Figures 1A,B. The results showed that the plasma Aβ42, Aβ40, and P-Tau levels of the AD group were higher than those of the NC group (*P* < 0.05), and the plasma Aβ40/Aβ42 of the AD group was lower than that of the NC group (*P* < 0.05); plasma Aβ42, Aβ40, and P-Tau levels in the AD group were higher than those in the aMCI group (*P* < 0.05), and plasma Aβ40/Aβ42 in the AD group was lower than that in the aMCI group, but the difference was not statistically significant (*P* > 0.05); plasma P-Tau and Aβ42 showed statistical difference (*P* < 0.05), while there was no statistical difference in plasma Aβ40 and Aβ40/Aβ42 levels between the aMCI and NC groups (*P* > 0.05).

### Surface-Based Morphometry Data Result Statistics

In the ANOVA comparison, the regions with differences in the cortical thickness of the three groups included the right superior temporal gyrus, temporal pole, entorhinal cortex, transverse temporal gyrus, insula, fusiform gyrus, superior marginal gyrus, middle temporal gyrus, superior temporal gyrus (cluster 1, *F* = 16.4, *P* < 0.001), and posterior cingulate gyrus (cluster 2, *F* = 11.1, *P* < 0.001).

**FIGURE 2:**
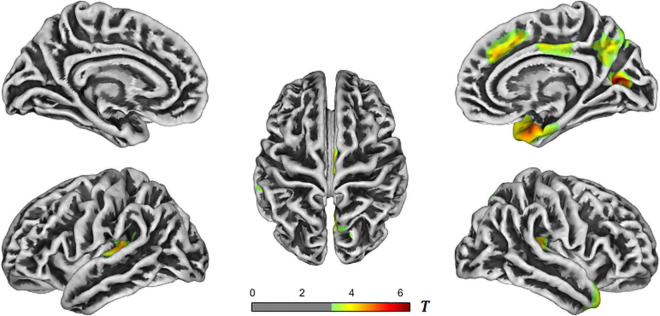
*T*-value maps of brain regions with reduced cortical thickness in the AD-NC groups. The closer the color mark was to red, the more severe the atrophy [threshold-free cluster enhancement (TFCE) multiple comparison correction, *P* < 0.05]. AD, Alzheimer’s disease; NC, normal control.

Further comparison between the groups were conducted. In the AD-NC groups, the brain regions with reduced cortical thickness mainly included the bilateral superior temporal gyrus, transverse temporal gyrus, bilateral superior marginal gyrus, bilateral insula, right temporal pole, right entorhinal cortex, right fusiform gyrus, right superior parietal lobule, right precuneus, right cuneus, right superior frontal gyrus, and right cingulate gyrus. See Table 2 and Figure 2 for details.

**TABLE 2:** Brain regions with reduced cortical thickness in the AD-NC groups.

	*T*-value	Size	Mean cortical thickness in AD group (mm)	Mean cortical thickness in NC group (mm)	Overlap of atlas (%)	Region
Cluster 1	4.6	435	2.22 ± 0.14	2.40 ± 0.17	40	Left transverse temporal
			–	–	39	Left superior temporal
			–	–	13	Left insula
			–	–	8	Left supramarginal
Cluster 2	5.0	1128	2.73 ± 0.24	3.04 ± 0.20	36	Right superior temporal
			–	–	14	Right temporal pole
			–	–	13	Right entorhinal
			–	–	11	Right transverse temporal
			–	–	10	Right insula
			–	–	6	Right supramarginal
			–	–	5	Right fusiform
Cluster 3	4.1	437	2.45 ± 0.19	2.64 ± 0.12	81	Right precuneus
			–	–	19	Right superior parietal
Cluster 4	5.8	283	1.77 ± 0.10	1.90 ± 0.09	65	Right pericalcarine
			–	–	29	Right cuneus
			–	–	6	Right precuneus
Cluster 5	4.4	272	2.82 ± 0.20	3.04 ± 0.20	100	Right superior frontal
Cluster 6	4.0	254	2.65 ± 0.15	2.82 ± 0.15	100	Right posterior cingulate

*AD, Alzheimer’s disease; NC, normal control.*

**FIGURE 3:**
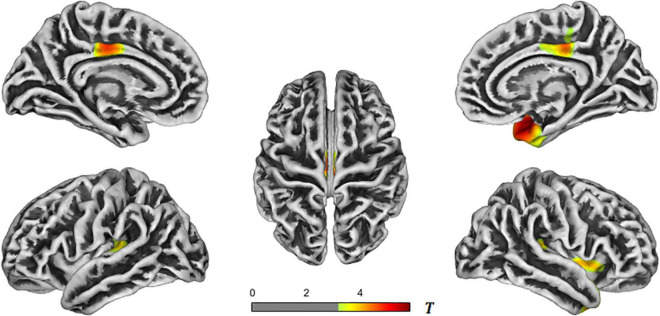
*T*-value maps of brain regions with reduced cortical thickness in the AD-aMCI groups. The closer the color mark was to red, the more severe the atrophy [threshold-free cluster enhancement (TFCE) multiple comparison correction, *P* < 0.05]. AD, Alzheimer’s disease; aMCI, amnestic mild cognitive impairment.

In the AD-aMCI groups, the brain regions with reduced cortical thickness primarily included the bilateral superior temporal gyrus, bilateral transverse temporal gyrus, bilateral insula, bilateral posterior cingulate gyrus, right temporal pole, right entorhinal cortex, right fusiform gyrus, and right paracentral gyrus. See Table 3 and Figure 3 for details. The aMCI and NC groups were processed with the same correction method, and the results showed no statistical difference in brain regions.

**TABLE 3:** Brain regions with reduced cortical thickness in the AD-aMCI groups.

	*T*-value	Size	Mean cortical thickness in AD group (mm)	Mean cortical thickness in aMCI group (mm)	Overlap of atlas (%)	Region
Cluster 1	4.4	276	2.29 ± 0.17	2.47 ± 0.16	51	Left transverse temporal
			–	–	30	Left insula
			–	–	16	Left superior temporal
Cluster 2	4.8	196	2.56 ± 0.17	2.74 ± 0.14	100	Left posterior cingulate
Cluster 3	5.3	1136	2.92 ± 0.26	3.21 ± 0.20	34	Right superior temporal
			–	–	26	Right insula
			–	–	14	Right temporal pole
			–	–	9	Right transverse temporal
			–	–	6	Right fusiform
			–	–	5	Right entorhinal
Cluster 4	4.6	346	2.54 ± 0.13	2.70 ± 0.12	80	Right posterior cingulate
			–	–	20	Right paracentral

*AD, Alzheimer’s disease; aMCI, amnestic mild cognitive impairment.*

### Correlation Analysis

We analyzed the correlation between the obtained whole-brain cortical thickness and the corresponding MMSE and MoCA scores to obtain relevant clusters, and extracted the cortical thickness of each cluster. It was found that the cortical thickness was positively correlated with the MMSE and MoCA scores. In other words, as the cortical thickness decreased, the MMSE and MoCA scores also decreased. See Table 4 for details.

**TABLE 4:** Correlation between cortical thickness and MMSE and MoCA scores.

		Cortical thickness									
		R					L				
		Cluster 1	Cluster 2	Cluster 3	Cluster 4	Cluster 5	Cluster 1	Cluster 2	Cluster 3	Cluster 4	Cluster 5
MMSE	*r*	0.539**	0.453**	0.421**	0.387**	0.382**	0.436**	0.457**	0.431**	0.435**	0.448**
	*P*	0.001	0.001	0.001	0.001	0.001	0.001	0.001	0.001	0.001	0.001
MoCA	*r*	0.535**	0.418**	0.396**	0.436**	–	0.420**	0.444**	0.410**	–	–
	*P*	0.001	0.001	0.001	0.001	–	0.001	0.001	0.001	–	–

MMSE, the Mini-Mental State Examination; MoCA, the Montreal Cognitive Assessment; R, right; L, left. **P < 0.001.

The cortical thickness of each cluster in the AD-NC groups and AD-aMCI groups was extracted with plasma Aβ40, Aβ42, and P-Tau for correlation analysis. The results showed that the cortical thickness of each cluster was negatively correlated with plasma Aβ40, Aβ42, and P-Tau. In other words, as the cortical thickness decreased, the plasma Aβ40, Aβ42, and P-Tau values increased. See Table 5 for details.

**TABLE 5:** Correlation between cortical thickness and plasma Aβ and Tau.

		Tau		Aβ42		Aβ40		Aβ42/40	
		*r*	*P*	*r*	*P*	*r*	*P*	*r*	*P*
AD-NC	Cluster 1R	−0.346**	0.005	−0.439**	0.001	−0.322**	0.009	0.202	0.11
	Cluster 2R	–0.177	0.161	−0.282*	0.024	–0.129	0.308	0.049	0.702
	Cluster 3R	−0.249*	0.047	−0.305*	0.014	–0.179	0.157	0.109	0.389
	Cluster 4R	−0.246*	0.05	−0.296*	0.017	–0.132	0.299	0.204	0.105
	Cluster 5R	−0.301*	0.016	−0.252*	0.044	−0.385**	0.002	0.233	0.064
	Cluster 1L	−0.312*	0.012	−0.336**	0.007	−0.261*	0.037	0.198	0.118
AD-aMCI	Cluster 1R	–0.175	0.122	−0.296**	0.008	–0.208	0.066	0.075	0.51
	Cluster 2R	−0.290**	0.01	−0.298**	0.008	−0.305**	0.006	0.109	0.341
	Cluster 1L	–0.208	0.065	−0.239*	0.034	−0.224*	0.047	0.075	0.51
	Cluster 2L	−0.231*	0.04	−0.278*	0.013	–0.184	0.104	0.089	0.433

AD, Alzheimer’s disease; aMCI, amnestic mild cognitive impairment; NC, normal control; R, right; L, left. *P <0.05, **P < 0.01.

### Regression Analysis and Receiver Operating Characteristic Curves

Plasma Tau, Aβ40, and Aβ42, and the cortical thickness of each cluster with statistical difference in the AD-NC groups and AD-aMCI groups were subjected to multivariate ordered logistic analysis, and the results were as follows: ordered logistic regression in line with the proportional odds assumption was applied to analyze the influence of plasma Tau, Aβ40, and Aβ42 and the cortical thickness of each cluster on the severity of the patients’ disease.

The result of the score test for the proportional odds assumption (χ^2^ = 22.33, *P* = 0.051) indicated that the proportional odds assumption existed. The deviance goodness-of-fit test showed that the model fitted well (χ^2^ = 136.42, *P* > 0.999). The model goodness-of-fit test illustrated that this model was better than the model with only constant terms (χ^2^ = 105.213, *P* < 0.001). Four regression independent variables of P-Tau, Aβ40, Aβ42, and AD-NC cluster 1R were selected through screening. For every 1-unit increase in P-Tau, the OR value of “disease severity” increased by 0.036 times (95% CI: 1.018–1.055, χ^2^ = 15.984, *P* < 0.001). For every 1-unit increase in Aβ42, the OR value of “risk of progression to AD” elevated by 0.019 times (95% CI: 1.004–1.033, χ^2^ = 6.669, *P* = 0.01). For every 1-unit increase in Aβ40, the OR value of “disease severity” increased by 0.013 times (95% CI: 1.000–1.027, χ^2^ = 3.741, *P* = 0.053). For every 1-unit decrease in AD-NC cluster 1R, the OR value of “risk of progression to AD” decreased by 99.7% (95% CI: 7.87E^–06^–1.078, χ^2^ = 3.744, *P* = 0.05).

**FIGURE 4:**
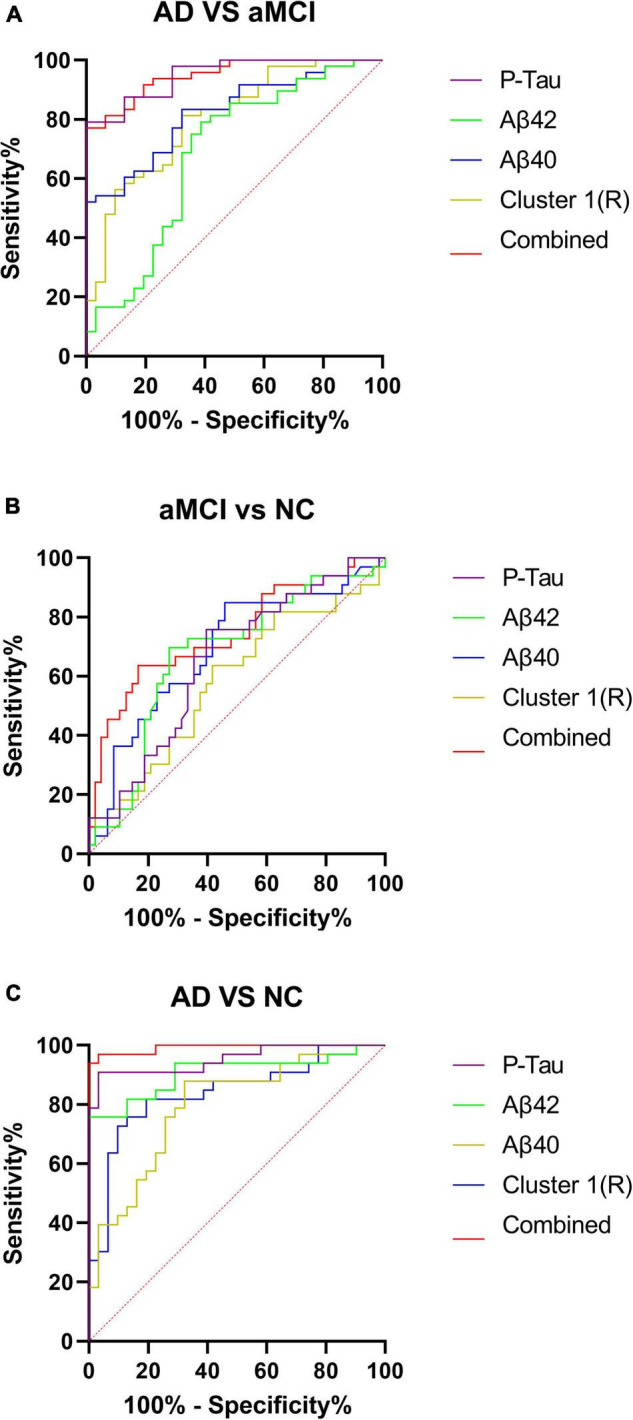
**(A–C)** Receiver operating characteristic curves for the AD-aMCI, aMCI-NC, and AD-NC groups. AD, Alzheimer’s disease; aMCI, amnestic mild cognitive impairment; NC, normal control.

Four regression independent variables of P-Tau, Aβ40, Aβ42, and AD-NC cluster 1R were selected by multiple ordered regression analysis. The above four independent variables were used to plot the ROC curves of the AD-aMCI, aMCI-NC, and AD-NC groups, respectively, and combined ROC curves were plotted for these four variables. The results were shown in Figures 4A–C.

## Discussion

### Analysis of Plasma Aβ42, Aβ40, and Phosphorylated Tau Results

We analyzed plasma Aβ40, Aβ42, and P-Tau concentrations in the AD, aMCI, and NC groups, and found that with the progression of the disease, plasma Aβ40, Aβ42, and P-Tau in the three groups showed upward trends, which was consistent with the findings of Yang et al. (2018) and Zecca et al. (2018). Although the change trend of plasma Aβ and Tau is still controversial, many previous studies confirmed that the change trend of plasma Aβ and Tau could reflect the progress of AD disease to a certain extent (Nakamura et al., 2018; Chatterjee et al., 2019; Chen et al., 2019; Risacher et al., 2019), and similar results were obtained in this study.

This may be attributed to a large amount of Aβ and Tau produced in the brain of AD patients. Most of Aβ and Tau are degraded by the ubiquitin-proteasome system and autophagy system in the brain (Tarasoff-Conway et al., 2015; Xin et al., 2018), or are cleared in the brain by proteases secreted by astrocytes and neurons, while remaining Aβ and Tau enter the peripheral blood circulation system through the blood–brain barrier, blood–CSF barrier, arachnoid villi, lymphatic drainage, etc. (Congdon and Sigurdsson, 2018; Gao et al., 2018), causing the concentration of Aβ and Tau in peripheral blood to increase. As the disease progresses, more Aβ and Tau are generated in the brain, so Aβ and Tau concentrations in the peripheral blood tend to increase with the progression of the disease. Teunissen et al. (2018) and Yang et al. (2018) also drew similar conclusions. In addition, Aβ in CSF can be transported to the peripheral blood circulation system through endocytosis of LPR-1 receptors on the membrane surfaces of the brain microvascular endothelial cells (BMECs) that constitute the blood–brain barrier structure and ATP-dependent pump mediated by P-glycoprotein (P-gp). Although Tau protein lacks specific binding proteins to pass through the blood–brain barrier, Tau can still exchange substances with the peripheral blood circulation system through the above other pathways except the blood–brain barrier pathway. The above pathways all ensure that Aβ and Tau in the brain can be timely transported to the periphery blood for clearance, which may also be the cause of increases in Aβ and P-Tau concentrations in the peripheral blood. However, the correlation between Aβ40, Aβ42, and P-Tau in the peripheral blood and cortical thickness is not great. The analysis may be related to the sensitivity of the ELISA detection method to the lowest detection threshold of plasma Aβ40, Aβ42, and P-Tau. Therefore, more sensitive detection methods for Aβ40, Aβ42, and P-Tau in the peripheral blood as early diagnostic approaches for AD still require a lot of explorations.

### Analysis of Cortical Thickness Results

In the AD-NC groups, six clusters of brain regions with reduced cortical thickness were obtained, located in the bilateral superior temporal gyrus, transverse temporal gyrus, bilateral superior marginal gyrus, bilateral insula, right temporal pole, right entorhinal cortex, right fusiform, right superior parietal lobule, right precuneus, right cuneus, right superior frontal gyrus, and right cingulate gyrus. In the AD-aMCI groups, four clusters of brain regions with reduced cortical thickness were obtained, located in the bilateral superior temporal gyrus, bilateral transverse temporal gyrus, bilateral insula, bilateral posterior cingulate gyrus, right temporal pole, right entorhinal cortex, right fusiform gyrus, and right paracentral gyrus. It could be seen that the regions of brain atrophy showed expanding trends with the progress of AD disease. Many previous studies (Dickerson et al., 2009; Sabuncu et al., 2011; Wang et al., 2015) also validated that brain atrophy in AD patients mainly occurred in the entorhinal cortex, temporal pole, inferior temporal gyrus, middle temporal gyrus, superior parietal lobule, inferior parietal lobule, posterior cingulate gyrus, and other brain regions. The brain atrophy regions obtained in this study were consistent with the above research results, verifying that as the disease progressed in AD patients, the development of brain atrophy showed a trend of global brain atrophy.

### Correlation Analysis of Cortical Thickness and Mini-Mental State Examination, Montreal Cognitive Assessment Scale, and Plasma Aβ42, Aβ40, and Phosphorylated Tau Results

Through the correlation analysis of cortical thickness and MMSE and MoCA scale scores, correlated brain regions were obtained, respectively. Then the common brain regions related to MMSE and MoCA scores were further compared and found that the common brain regions primarily included the superior temporal gyrus, transverse temporal gyrus, entorhinal cortex, fusiform gyrus, parahippocampal gyrus, middle orbital frontal gyrus, superior frontal gyrus, middle frontal gyrus, frontal pole, and other brain regions. The above-mentioned brain regions such as the hippocampus, parahippocampal gyrus, entorhinal cortex, cingulate gyrus, anterior nucleus of the thalamus, insula, and frontal orbital surface are also an important part of the Papez circuit or limbic system, which is closely related to learning and memory (Sitoh and Tien, 1997; Aggleton et al., 2016), and emit complex and closely connected fiber projections among various structures to participate in the whole process of learning and memory. This correlation indicated the importance of these brain regions in each cluster for the diagnosis of AD disease.

At present, Aβ and Tau in the brain are mainly determined by Pittsburgh compound B positron emission tomography (PiB-PET), flortaucipir (FTP)-PET, and CSF examination. Aβ and Tau content in the brain is analyzed with cortical thickness to assess their correlation, and a large number of studies supported the negative correlation between Aβ and Tau in the brain and cortical thickness or gray matter volume (Hsu et al., 2017; Pereira et al., 2017; Digma et al., 2019; Ossenkoppele et al., 2019; Harrison et al., 2021). In this study, correlation analysis was performed between the cortical thickness results and plasma Aβ42, Aβ40, and P-Tau concentration results. It was shown that the cortical thickness values of each cluster were also negatively correlated with plasma Aβ40, Aβ42, and P-Tau, which was consistent with the correlation between Aβ and Tau in the brain and changes in cortical thickness, suggesting that Aβ and Tau in the peripheral blood can reflect changes in Aβ and Tau in the brain to a certain extent. However, this study was a cross-sectional study, and each study cohort was limited to subjects who were clinically evaluated. The results still need to be confirmed by longitudinal study and analysis with a larger sample.

### Regression Analysis and Receiver Operating Characteristic Curves

In this study, multivariate ordered logistic analysis was used to screen plasma indexes and the cortical thickness of each cluster with statistical differences in the AD-NC groups and AD-aMCI groups. Finally, plasma Aβ40, Aβ42, P-Tau, and AD-NC cluster 1R (right superior temporal gyrus, temporal pole, entorhinal cortex, transverse temporal gyrus, fusiform gyrus, superior marginal gyrus, middle temporal gyrus, and inferior temporal gyrus) were obtained. The brain regions involved in clusters obtained from the above screening revealed that the brain regions with reduced cortical thickness were mainly concentrated in the temporal lobe, which is closely related to the progression of MCI and AD. The selected factors were further used to draw ROC curves of the AD-NC, AD-aMCI, and aMCI-NC groups. See Table 6 for details. It was found that the sensitivity, specificity and the area under the curve of plasma Aβ40, Aβ42, and P-Tau were all greater than cortical thickness indexes in the AD-NC, AD-aMCI, and aMCI-NC groups, certifying that plasma Aβ40, Aβ42, and P-Tau had good sensitivity and specificity for the early recognition of AD, which was in agreement with the studies of Schindler et al. (2019), Shen et al. (2020), and Zou et al. (2020). Plasma Aβ40, Aβ42, and P-Tau are still expected to be applied in the future as biological indexes for the early recognition of AD. Plasma indexes and cortical thickness indexes in the AD-NC, AD-aMCI, and aMCI-NC groups were combined to plot ROC curves. The diagnostic efficiency of the combined curve of each group was better than that of plasma or cortical thickness indexes alone, which was supported by Rahim et al. (2017), Zhang and Liu (2018), and Gupta et al. (2019), suggesting that the early recognition and diagnosis of AD still need to be combined with multiple factors for analysis.

**TABLE 6:** Receiver operating characteristic curve parameters of the AD-NC, AD-aMCI, and aMCI-NC groups.

		Area	SE	95% confidence interval	*P*
AD-NC	P-Tau	0.953	0.026	0.902–1.000	<0.0001
	AB42	0.907	0.040	0.829–0.986	<0.0001
	AB40	0.795	0.056	0.684–0.905	<0.0001
	Cluster 1R	0.841	0.051	0.740–0.941	<0.0001
	Combined	0.992	0.008	0.977–1.000	<0.0001
AD-aMCI	P-Tau	0.950	0.021	0.908–0.991	<0.0001
	AB42	0.684	0.065	0.557–0.811	0.0059
	AB40	0.822	0.046	0.732–0.912	<0.0001
	Cluster 1R	0.800	0.050	0.702–0.899	<0.0001
	Combined	0.948	0.022	0.906–0.991	<0.0001
aMCI-NC	P-Tau	0.654	0.061	0.534–0.774	0.0190
	AB42	0.674	0.062	0.552–0.796	0.0081
	AB40	0.690	0.061	0.571–0.810	0.0038
	Cluster 1R	0.581	0.066	0.452–0.709	0.2186
	Combined	0.745	0.058	0.632–0.858	0.0002

*AD, Alzheimer’s disease; aMCI, amnestic mild cognitive impairment; NC, normal control.*

This study found that AD patients developed cortical atrophy characterized by atrophy of the medial temporal lobe with the progression of the disease. Plasma Aβ40, Aβ42, and P-Tau were negatively correlated with cortical thickness, this suggests that the decrease of cortical thickness and the increase of plasma A concentration may indicate the progression of dementia to some extent. Additionally, this study also revealed that combined plasma and cortical thickness indicators can better identify AD.

## Data Availability Statement

The raw data supporting the conclusions of this article will be made available by the authors, without undue reservation.

## Ethics Statement

The studies involving human participants were reviewed and approved by the Ethics Committee of the Affiliated Zhongshan Hospital of Dalian University (approval number: 2019142). The patients/participants provided their written informed consent to participate in this study. Written informed consent was obtained from the individual(s) for the publication of any potentially identifiable images or data included in this article.

## Author Contributions

KL and QM designed the study. KL wrote the manuscript. HQ, MM, CX, MC, FH, QZ, and XG collected, analyzed, and interpreted the data. QM critically reviewed, edited, and approved the manuscript. All authors read and approved the final manuscript.

## Conflict of Interest

The authors declare that the research was conducted in the absence of any commercial or financial relationships that could be construed as a potential conflict of interest.

## Publisher’s Note

All claims expressed in this article are solely those of the authors and do not necessarily represent those of their affiliated organizations, or those of the publisher, the editors and the reviewers. Any product that may be evaluated in this article, or claim that may be made by its manufacturer, is not guaranteed or endorsed by the publisher.

## Footnotes

1
https://www.fil.ion.ucl.ac.uk/spm/software/spm12/

2
http://www.neuro.uni-jena.de/cat12/

3
https://www.mathworks.com/

## References

[B1] Aggleton J. P. Pralus A. Nelson A. J. Hornberger M. (2016). Thalamic pathology and memory loss in early Alzheimer’s disease: moving the focus from the medial temporal lobe to Papez circuit. *Brain* 139 1877–1890. 10.1093/brain/aww083 27190025 PMC4939698

[B2] Arthur G. L. Brende J. O. Locicero K. A. (2001). *Diagnostic and statistical manual of mental disorders (4th ed) and text revision.* Virginia: American Psychiatric Association.

[B3] Berg L. (1988). Clinical dementia rating (CDR). *Psychopharmacol. Bull.* 24 637–639. 3249765

[B4] Chatterjee P. Elmi M. Goozee K. Shah T. Sohrabi H. R. Dias C. B. (2019). Ultrasensitive detection of plasma Amyloid-β as a biomarker for cognitively normal elderly individuals at risk of alzheimer’s disease. *J. Alzheimers Dis.* 71 775–783. 10.3233/jad-190533 31424403

[B5] Chen T. B. Lee Y. J. Lin S. Y. Chen J. P. Hu C. J. Wang P. N. (2019). Plasma Aβ42 and total tau predict cognitive decline in amnestic mild cognitive impairment. *Sci. Rep.* 9:13984. 10.1038/s41598-019-50315-9 31562355 PMC6764975

[B6] Congdon E. E. Sigurdsson E. M. (2018). Tau-targeting therapies for Alzheimer disease. *Nat. Rev. Neurol.* 14 399–415. 10.1038/s41582-018-0013-z 29895964 PMC6463489

[B7] Dahnke R. Yotter R. A. Gaser C. (2013). Cortical thickness and central surface estimation. *Neuroimage* 65 336–348. 10.1016/j.neuroimage.2012.09.050 23041529

[B8] Desikan R. S. Ségonne F. Fischl B. Quinn B. T. Dickerson B. C. Blacker D. (2006). An automated labeling system for subdividing the human cerebral cortex on MRI scans into gyral based regions of interest. *Neuroimage* 31 968–980. 10.1016/j.neuroimage.2006.01.021 16530430

[B9] Dickerson B. C. Bakkour A. Salat D. H. Feczko E. Pacheco J. Greve D. N. (2009). The cortical signature of Alzheimer’s disease: regionally specific cortical thinning relates to symptom severity in very mild to mild AD dementia and is detectable in asymptomatic amyloid-positive individuals. *Cereb. Cortex.* 19 497–510. 10.1093/cercor/bhn113 18632739 PMC2638813

[B10] Digma L. A. Madsen J. R. Reas E. T. Dale A. M. Brewer J. B. Banks S. J. (2019). Tau and atrophy: domain-specific relationships with cognition. *Alzheimers Res. Ther.* 11:65. 10.1186/s13195-019-0518-8 31351484 PMC6661099

[B11] Dubois B. Feldman H. H. Jacova C. Dekosky S. T. Barberger-Gateau P. Cummings J. (2007). Research criteria for the diagnosis of Alzheimer’s disease: revising the NINCDS-ADRDA criteria. *Lancet Neurol.* 6 734–746. 10.1016/s1474-4422(07)70178-3 17616482

[B12] Dubois B. Feldman H. H. Jacova C. Hampel H. Molinuevo J. L. Blennow K. (2014). Advancing research diagnostic criteria for Alzheimer’s disease: the IWG-2 criteria. *Lancet Neurol.* 13 614–629. 10.1016/s1474-4422(14)70090-0 24849862

[B13] Folstein M. F. Folstein S. E. McHugh P. R. (1975). Mini-mental state”. A practical method for grading the cognitive state of patients for the clinician. *J. Psychiatr. Res.* 12 189–198. 10.1016/0022-3956(75)90026-6 1202204

[B14] Gao Y. Tan L. Yu J. T. Tan L. (2018). Tau in alzheimer’s disease: mechanisms and therapeutic strategies. *Curr. Alzheimer Res.* 15 283–300. 10.2174/1567205014666170417111859 28413986

[B15] Gupta Y. Lee K. H. Choi K. Y. Lee J. J. Kim B. C. Kwon G. R. (2019). Early diagnosis of Alzheimer’s disease using combined features from voxel-based morphometry and cortical, subcortical, and hippocampus regions of MRI T1 brain images. *PLoS One* 14:e0222446. 10.1371/journal.pone.0222446 31584953 PMC6777799

[B16] Hamilton M. (1959). The assessment of anxiety states by rating. *Br. J. Med. Psychol.* 32 50–55. 10.1111/j.2044-8341.1959.tb00467.x 13638508

[B17] Hamilton M. (1960). A rating scale for depression. *J. Neurol. Neurosurg. Psychiatry* 23 56–62. 10.1136/jnnp.23.1.56 14399272 PMC495331

[B18] Harrison T. M. Du R. Klencklen G. Baker S. L. Jagust W. J. (2021). Distinct effects of beta-amyloid and tau on cortical thickness in cognitively healthy older adults. *Alzheimers Dement.* 17 1085–1096. 10.1002/alz.12249 33325068 PMC8203764

[B19] Horínek D. Varjassyová A. Hort J. (2007). Magnetic resonance analysis of amygdalar volume in Alzheimer’s disease. *Curr. Opin. Psychiatry* 20 273–277. 10.1097/YCO.0b013e3280ebb613 17415082

[B20] Hsu J. L. Lee W. J. Liao Y. C. Lirng J. F. Wang S. J. Fuh J. L. (2017). Plasma biomarkers are associated with agitation and regional brain atrophy in Alzheimer’s disease. *Sci. Rep.* 7:5035. 10.1038/s41598-017-05390-1 28698646 PMC5506051

[B21] Jack C. R. Jr. Albert M. S. Knopman D. S. McKhann G. M. Sperling R. A. Carrillo M. C. (2011). Introduction to the recommendations from the National Institute on Aging-Alzheimer’s Association workgroups on diagnostic guidelines for Alzheimer’s disease. *Alzheimers Dement.* 7 257–262. 10.1016/j.jalz.2011.03.004 21514247 PMC3096735

[B22] Jack C. R. Jr. Knopman D. S. Jagust W. J. Petersen R. C. Weiner M. W. Aisen P. S. (2013). Tracking pathophysiological processes in Alzheimer’s disease: an updated hypothetical model of dynamic biomarkers. *Lancet. Neurol.* 12, 207–216. 10.1016/s1474-4422(12)70291-0 23332364 PMC3622225

[B23] Liu P. P. Xie Y. Meng X. Y. Kang J. S. (2019). History and progress of hypotheses and clinical trials for Alzheimer’s disease. *Signal. Transduct. Target Ther.* 4:29. 10.1038/s41392-019-0063-8 31637009 PMC6799833

[B24] Lue L. F. Sabbagh M. N. Chiu M. J. Jing N. Snyder N. L. Schmitz C. (2017). Plasma Levels of Aβ42 and tau identified probable alzheimer’s dementia: findings in two cohorts. *Front. Aging Neurosci.* 9:226. 10.3389/fnagi.2017.00226 28790911 PMC5522888

[B25] Maass A. Lockhart S. N. Harrison T. M. Bell R. K. Mellinger T. Swinnerton K. (2018). Entorhinal tau pathology, episodic memory decline, and neurodegeneration in aging. *J. Neurosci.* 38 530–543. 10.1523/jneurosci.2028-17.2017 29192126 PMC5777108

[B26] Marquié M. Siao Tick Chong M. Antón-Fernández A. Verwer E. E. Sáez-Calveras N. Meltzer A. C. (2017). [F-18]-AV-1451 binding correlates with postmortem neurofibrillary tangle Braak staging. *Acta Neuropathol.* 134 619–628. 10.1007/s00401-017-1740-8 28612291 PMC5772971

[B27] Morris J. C. (1993). The Clinical Dementia Rating (CDR): current version and scoring rules. *Neurology* 43 2412–2414. 10.1212/wnl.43.11.2412-a 8232972

[B28] Moscoso A. Silva-Rodríguez J. Aldrey J. M. Cortés J. Fernández-Ferreiro A. Gómez-Lado N. (2019). Prediction of Alzheimer’s disease dementia with MRI beyond the short-term: Implications for the design of predictive models. *Neuroim. Clin.* 23:101837. 10.1016/j.nicl.2019.101837 31078938 PMC6515129

[B29] Nakamura A. Kaneko N. Villemagne V. L. Kato T. Doecke J. Dore V. (2018). High performance plasma amyloid-β biomarkers for Alzheimer’s disease. *Nature* 554 249–254. 10.1038/nature25456 29420472

[B30] Nasreddine Z. S. Phillips N. A. Bedirian V. Charbonneau S. Whitehead V. Collin I. (2005). The Montreal Cognitive Assessment, MoCA: a brief screening tool for mild cognitive impairment. *J. Am. Geriatr. Soc.* 53 695–699. 10.1111/j.1532-5415.2005.53221.x 15817019

[B31] Ossenkoppele R. Smith R. Ohlsson T. Strandberg O. Mattsson N. Insel P. S. (2019). Associations between tau, Aβ, and cortical thickness with cognition in Alzheimer disease. *Neurology* 92 e601–e612. 10.1212/wnl.0000000000006875 30626656 PMC6382060

[B32] Palmqvist S. Insel P. S. Stomrud E. Janelidze S. Zetterberg H. Brix B. (2019). Cerebrospinal fluid and plasma biomarker trajectories with increasing amyloid deposition in Alzheimer’s disease. *EMBO Mol. Med.* 11:e11170. 10.15252/emmm.201911170 31709776 PMC6895602

[B33] Pereira J. B. Westman E. Hansson O. Alzheimer’s Disease Neuroimaging I. (2017). Association between cerebrospinal fluid and plasma neurodegeneration biomarkers with brain atrophy in Alzheimer’s disease. *Neurobiol. Aging* 58 14–29. 10.1016/j.neurobiolaging.2017.06.002 28692877

[B34] Petersen R. C. (2004). Mild cognitive impairment as a diagnostic entity. *J. Intern. Med.* 256 183–194. 10.1111/j.1365-2796.2004.01388.x 15324362

[B35] Petersen R. C. Smith G. E. Waring S. C. Ivnik R. J. Tangalos E. G. Kokmen E. (1999). Mild cognitive impairment: clinical characterization and outcome. *Arch. Neurol.* 56 303–308. 10.1001/archneur.56.3.303 10190820

[B36] Pettigrew C. Soldan A. Zhu Y. Wang M. C. Brown T. Miller M. (2017). Cognitive reserve and cortical thickness in preclinical Alzheimer’s disease. *Brain Imag. Behav.* 11 357–367. 10.1007/s11682-016-9581-y 27544202 PMC5743433

[B37] Pettigrew C. Soldan A. Zhu Y. Wang M. C. Moghekar A. Brown T. (2016). Cortical thickness in relation to clinical symptom onset in preclinical AD. *Neuroimag. Clin.* 12 116–122. 10.1016/j.nicl.2016.06.010 27408796 PMC4932610

[B38] Portet F. Ousset P. J. Visser P. J. Frisoni G. B. Nobili F. Scheltens P. (2006). Mild cognitive impairment (MCI) in medical practice: a critical review of the concept and new diagnostic procedure. Report of the MCI Working Group of the European Consortium on Alzheimer’s Disease. *J. Neurol. Neurosurg. Psychiatry* 77 714–718. 10.1136/jnnp.2005.085332 16549412 PMC2077456

[B39] Racine A. M. Brickhouse M. Wolk D. A. Dickerson B. C. (2018). The personalized Alzheimer’s disease cortical thickness index predicts likely pathology and clinical progression in mild cognitive impairment. *Alzheimers Dement.* 10 301–310. 10.1016/j.dadm.2018.02.007 29780874 PMC5956936

[B40] Rahim M. Thirion B. Bzdok D. Buvat I. Varoquaux G. (2017). Joint prediction of multiple scores captures better individual traits from brain images. *Neuroimage* 158 145–154. 10.1016/j.neuroimage.2017.06.072 28676298

[B41] Risacher S. L. Fandos N. Romero J. Sherriff I. Pesini P. Saykin A. J. (2019). Plasma amyloid beta levels are associated with cerebral amyloid and tau deposition. *Alzheimers Dement.* 11 510–519. 10.1016/j.dadm.2019.05.007 31384662 PMC6661419

[B42] Sabuncu M. R. Desikan R. S. Sepulcre J. Yeo B. T. Liu H. Schmansky N. J. (2011). The dynamics of cortical and hippocampal atrophy in Alzheimer disease. *Arch. Neurol.* 68 1040–1048. 10.1001/archneurol.2011.167 21825241 PMC3248949

[B43] Salimi-Khorshidi G. Smith S. M. Nichols T. E. (2011). Adjusting the effect of nonstationarity in cluster-based and TFCE inference. *Neuroimage* 54 2006–2019. 10.1016/j.neuroimage.2010.09.088 20955803

[B44] Schindler S. E. Bollinger J. G. Ovod V. Mawuenyega K. G. Li Y. Gordon B. A. (2019). High-precision plasma β-amyloid 42/40 predicts current and future brain amyloidosis. *Neurology* 93 e1647–e1659. 10.1212/wnl.0000000000008081 31371569 PMC6946467

[B45] Schneider L. (2020). A resurrection of aducanumab for Alzheimer’s disease. *Lancet Neurol.* 19 111–112. 10.1016/s1474-4422(19)30480-6 31978357

[B46] Sevigny J. Chiao P. Bussière T. Weinreb P. H. Williams L. Maier M. (2016). The antibody aducanumab reduces Aβ plaques in Alzheimer’s disease. *Nature* 537 50–56. 10.1038/nature19323 27582220

[B47] Shen X. N. Li J. Q. Wang H. F. Li H. Q. Huang Y. Y. Yang Y. X. (2020). Plasma amyloid, tau, and neurodegeneration biomarker profiles predict Alzheimer’s disease pathology and clinical progression in older adults without dementia. *Alzheimers Dement.* 12:e12104. 10.1002/dad2.12104 33005724 PMC7513626

[B48] Sitoh Y. Y. Tien R. D. (1997). The limbic system. An overview of the anatomy and its development. *Neuroimag. Clin. N. Am.* 7 1–10. 9100228

[B49] Sperling R. A. Aisen P. S. Beckett L. A. Bennett D. A. Craft S. Fagan A. M. (2011). Toward defining the preclinical stages of Alzheimer’s disease: recommendations from the National Institute on Aging-Alzheimer’s Association workgroups on diagnostic guidelines for Alzheimer’s disease. *Alzheimers Dement.* 7 280–292. 10.1016/j.jalz.2011.03.003 21514248 PMC3220946

[B50] Sun P. Lou W. Liu J. Shi L. Li K. Wang D. (2019). Mapping the patterns of cortical thickness in single- and multiple-domain amnestic mild cognitive impairment patients: a pilot study. *Aging* 11 10000–10015. 10.18632/aging.102362 31756169 PMC6914405

[B51] Tang X. Holland D. Dale A. M. Miller M. I. Alzheimer’s Disease Neuroimaging I. (2015). APOE Affects the volume and shape of the amygdala and the hippocampus in mild cognitive impairment and alzheimer’s disease: age matters. *J. Alzheimers Dis.* 47 645–660. 10.3233/JAD-150262 26401700 PMC5479937

[B52] Tarasoff-Conway J. M. Carare R. O. Osorio R. S. Glodzik L. Butler T. Fieremans E. (2015). Clearance systems in the brain-implications for Alzheimer disease. *Nat. Rev. Neurol.* 11 457–470. 10.1038/nrneurol.2015.119 26195256 PMC4694579

[B53] Teunissen C. E. Chiu M. J. Yang C. C. Yang S. Y. Scheltens P. Zetterberg H. (2018). Plasma Amyloid-β (Aβ42) Correlates with Cerebrospinal Fluid Aβ42 in Alzheimer’s Disease. *J. Alzheimers Dis.* 62 1857–1863. 10.3233/jad-170784 29614646

[B54] Wang L. Benzinger T. L. Hassenstab J. Blazey T. Owen C. Liu J. (2015). Spatially distinct atrophy is linked to β-amyloid and tau in preclinical Alzheimer disease. *Neurology* 84 1254–1260. 10.1212/wnl.0000000000001401 25716355 PMC4366088

[B55] Weise C. M. Bachmann T. Schroeter M. L. Saur D. (2019). When less is more: Structural correlates of core executive functions in young adults - A VBM and cortical thickness study. *Neuroimage* 189 896–903. 10.1016/j.neuroimage.2019.01.070 30716455

[B56] Xin S. H. Tan L. Cao X. Yu J. T. Tan L. (2018). Clearance of amyloid beta and tau in alzheimer’s disease: from mechanisms to therapy. *Neurotox. Res.* 34 733–748. 10.1007/s12640-018-9895-1 29626319

[B57] Yang C. C. Chiu M. J. Chen T. F. Chang H. L. Liu B. H. Yang S. Y. (2018). Assay of plasma phosphorylated tau protein (Threonine 181) and total tau protein in early-stage alzheimer’s disease. *J. Alzheimers Dis.* 61 1323–1332. 10.3233/JAD-170810 29376870

[B58] Zecca C. Tortelli R. Panza F. Arcuti S. Piccininni M. Capozzo R. (2018). Plasma β-amyloid(1-42) reference values in cognitively normal subjects. *J. Neurol. Sci.* 391 120–126. 10.1016/j.jns.2018.06.006 30103961

[B59] Zhang Y. Liu S. (2018). Analysis of structural brain MRI and multi-parameter classification for Alzheimer’s disease. *Biomed. Tech.* 63 427–437. 10.1515/bmt-2016-0239 28622141

[B60] Zhao Q. Guo Q. Liang X. Chen M. Zhou Y. Ding D. (2015). Auditory verbal learning test is superior to rey-osterrieth complex figure memory for predicting mild cognitive impairment to alzheimer’s disease. *Curr. Alzheimer Res.* 12 520–526. 10.2174/1567205012666150530202729 26027810

[B61] Zou K. Abdullah M. Michikawa M. (2020). Current biomarkers for alzheimer’s disease: from CSF to Blood. *J. Pers. Med.* 10:85. 10.3390/jpm10030085 32806668 PMC7564023

