# Supplementary material for: Correlation Between Brain Structure Atrophy and Plasma Amyloid-β and Phosphorylated Tau in Patients With Alzheimer’s Disease and Amnestic Mild Cognitive Impairment Explored by Surface-Based Morphometry
Source: Front Aging Neurosci. 2022 Apr 25;14:816043. doi: 10.3389/fnagi.2022.816043 (PMC9083065; doi:10.3389/fnagi.2022.816043)
Supplement: Supplementary file 1 [file Data_Sheet_1.PDF]

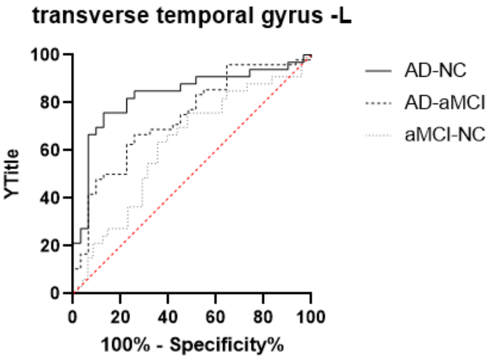
transverse temporal gyrus -L
YTitle
100% - Specificity%
0
20
40
60
80
100
AD-NC
AD-aMCI
aMCI-NC

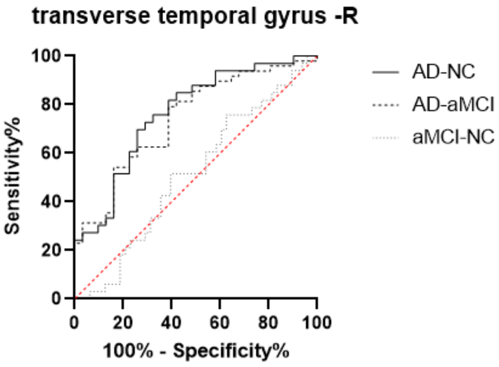
transverse temporal gyrus -R
Sensitivity%
100% - Specificity%
0
20
40
60
80
100
AD-NC
AD-aMCI
aMCI-NC

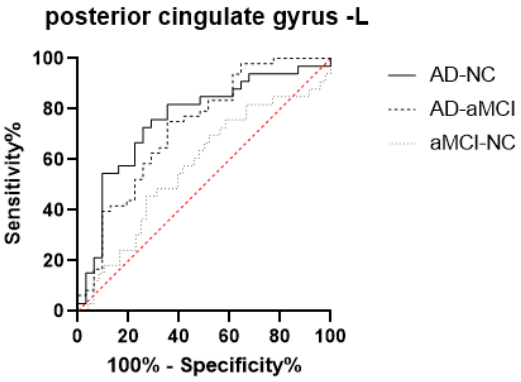
posterior cingulate gyrus -L
Sensitivity%
100% - Specificity%
0
20
40
60
80
100
AD-NC
AD-aMCI
aMCI-NC

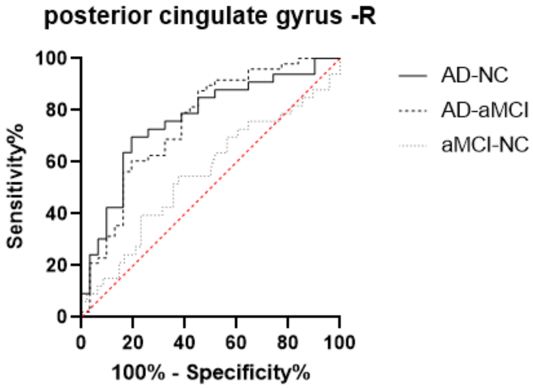
posterior cingulate gyrus -R
Sensitivity%
100% - Specificity%
0
20
40
60
80
100
AD-NC
AD-aMCI
aMCI-NC

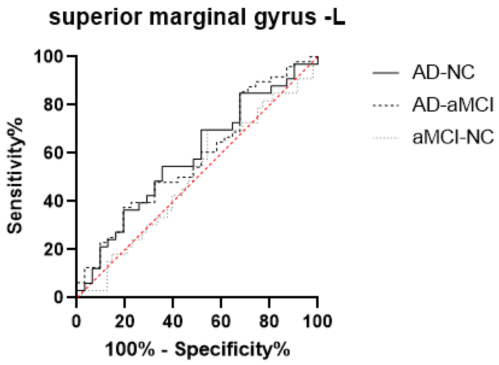
superior marginal gyrus -L
Sensitivity%
100% - Specificity%
0
20
40
60
80
100
AD-NC
AD-aMCI
aMCI-NC

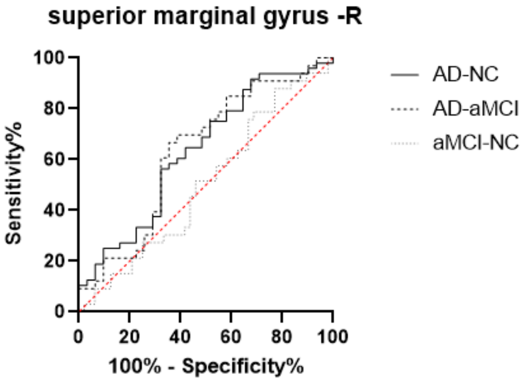
superior marginal gyrus -R
Sensitivity%
100% - Specificity%
0
20
40
60
80
100
AD-NC
AD-aMCI
aMCI-NC

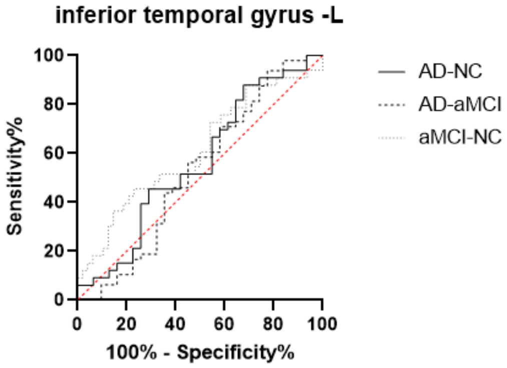
inferior temporal gyrus -L
Sensitivity%
100% - Specificity%
0
20
40
60
80
100
AD-NC
AD-aMCI
aMCI-NC

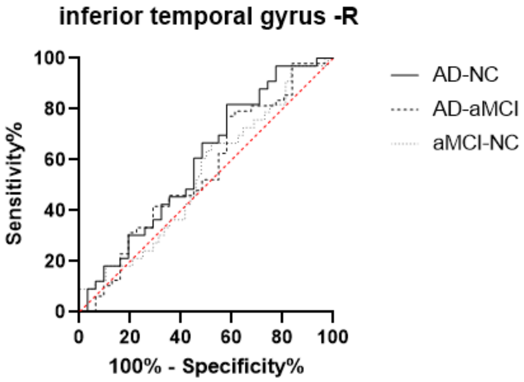
inferior temporal gyrus -R
Sensitivity%
100% - Specificity%
0
20
40
60
80
100
AD-NC
AD-aMCI
aMCI-NC

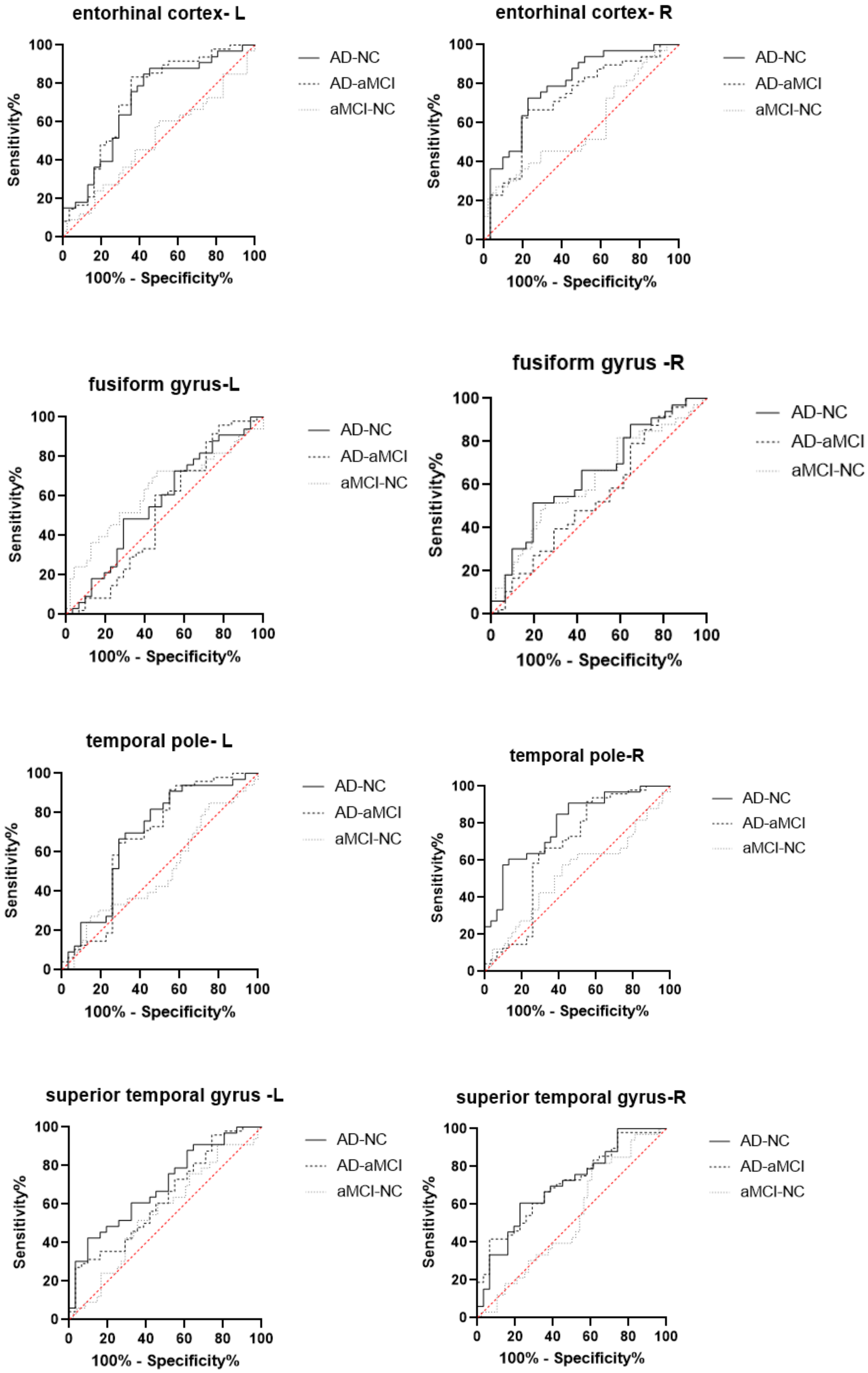
entorhinal cortex- L
entorhinal cortex- R
fusiform gyrus-L
fusiform gyrus -R
temporal pole- L
temporal pole-R
superior temporal gyrus -L
superior temporal gyrus-R
Sensitivity%
100% - Specificity%
0
20
40
60
80
100
AD-NC
AD-aMCI
aMCI-NC

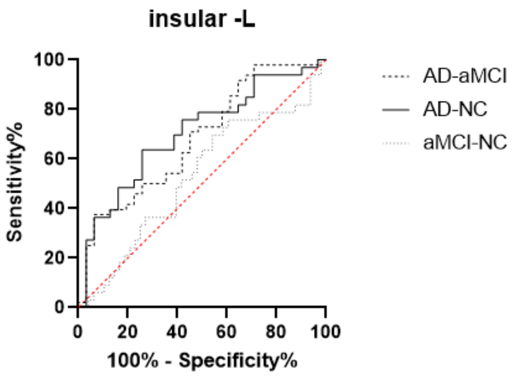
insular -L
Sensitivity%
100% - Specificity%
AD-aMCI
AD-NC
aMCI-NC

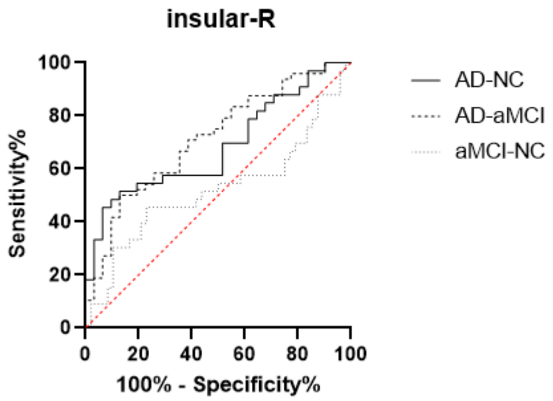
insular-R
Sensitivity%
100% - Specificity%
AD-NC
AD-aMCI
aMCI-NC

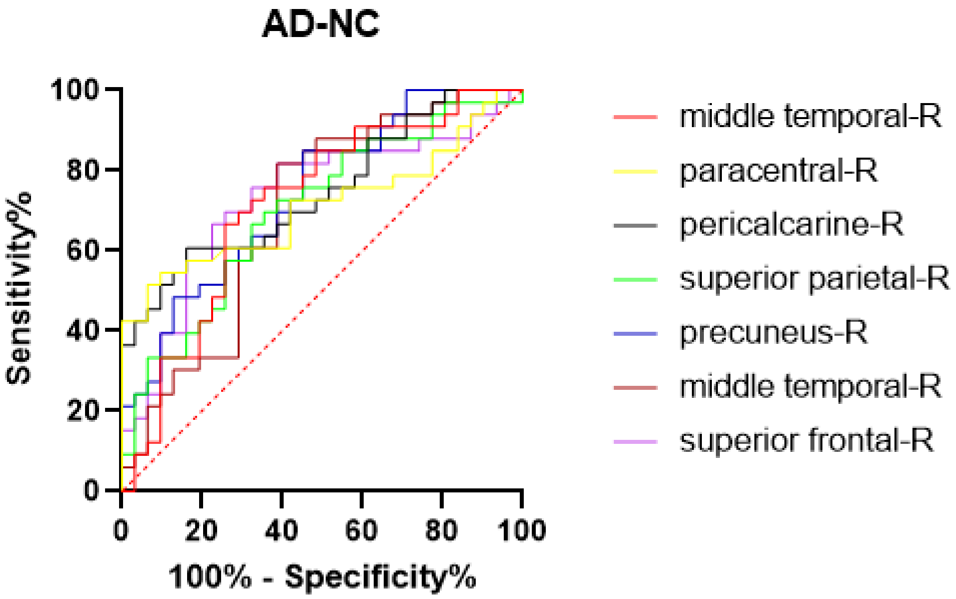
AD-NC
Sensitivity%
100% - Specificity%
middle temporal-R
paracentral-R
pericalcarine-R
superior parietal-R
precuneus-R
middle temporal-R
superior frontal-R

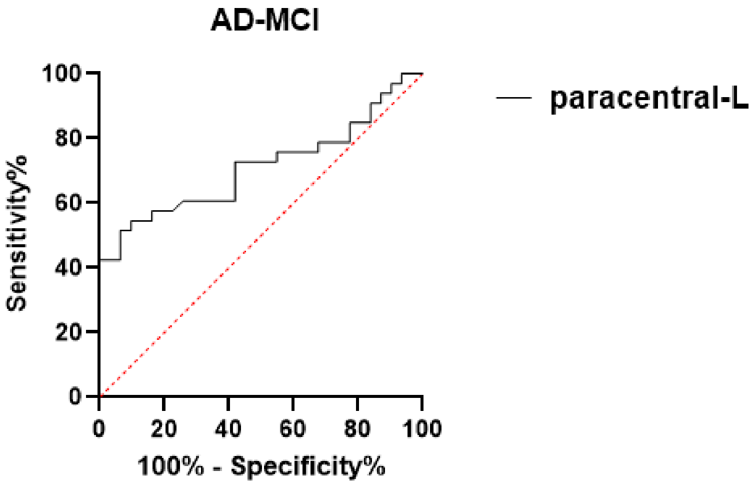
AD-MCI
Sensitivity%
100% - Specificity%
paracentral-L

We have tested different brain regions with Desikan-Killiany 40 Atlas. According to the segmentation of template, the cortical thickness of different brain regions with statistical differences in each group were extracted, and the cortical thickness of these brain regions was made into ROC curve according to AD-aMCI group, AD-NC group and aMCI-NC group. The result is as shown above, We found that the above brain regions are helpful for AD diagnosis, and select these brain regions as ROI(all the ROIs have been included in these clusters)，and multivariate ordered logistic regression analysis was used to screen out significant indexes, to obtain the diagnostic value of multi-factor combined prediction for cognitive dysfunction.
